# ICAM-1 suppresses tumor metastasis by inhibiting macrophage M2 polarization through blockade of efferocytosis

**DOI:** 10.1038/cddis.2015.144

**Published:** 2015-06-11

**Authors:** M Yang, J Liu, C Piao, J Shao, J Du

**Affiliations:** 1 Beijing Anzhen Hospital, Capital Medical University, Beijing, China; 2 Key Laboratory of Remodeling-Related Cardiovascular Diseases, Capital Medical University, Ministry of Education, Beijing Institute of Heart Lung and Blood Vessel Diseases, Beijing collaborative innovative research center for cardiovascular diseases, Beijing, China; 3 Second Affiliated Hospital to Nanchang University, Jiangxi 330006, China

## Abstract

Efficient clearance of apoptotic cells (efferocytosis) can profoundly influence tumor-specific immunity. Tumor-associated macrophages are M2-polarized macrophages that promote key processes in tumor progression. Efferocytosis stimulates M2 macrophage polarization and contributes to cancer metastasis, but the signaling mechanism underlying this process is unclear. Intercellular cell adhesion molecule-1 (ICAM-1) is a transmembrane glycoprotein member of the immunoglobulin superfamily, which has been implicated in mediating cell–cell interaction and outside-in cell signaling during the immune response. We report that ICAM-1 expression is inversely associated with macrophage infiltration and the metastasis index in human colon tumors by combining Oncomine database analysis and immunohistochemistry for ICAM-1. Using a colon cancer liver metastasis model in ICAM-1-deficient (ICAM-1^−/−^) mice and their wild-type littermates, we found that loss of ICAM-1 accelerated liver metastasis of colon carcinoma cells. Moreover, ICAM-1 deficiency increased M2 macrophage polarization during tumor progression. We further demonstrated that ICAM-1 deficiency in macrophages led to promotion of efferocytosis of apoptotic tumor cells through activation of the phosphatidylinositol 3 kinase/Akt signaling pathway. More importantly, coculture of ICAM-1^−/−^ macrophages with apoptotic cancer cells resulted in an increase of M2-like macrophages, which was blocked by an efferocytosis inhibitor. Our findings demonstrate a novel role for ICAM-1 in suppressing M2 macrophage polarization via downregulation of efferocytosis in the tumor microenvironment, thereby inhibiting metastatic tumor progression.

Macrophages have a central role in cancer development, as they constitute a substantial portion of the tumor mass and interact with numerous effector cells.^1, 2, 3, 4^ The role of macrophages in tumor progression has been shown to be double-edged, as these cells can both promote tumor rejection (M1 macrophages) and stimulate tumor growth (M2 macrophages). Pro-inflammatory, or classically activated M1 macrophages, exert important functions in host defense as well as tumoricidal activity, whereas anti-inflammatory, or alternatively activated M2 macrophages, have important roles in immune regulation, tissue remodeling, and tumor progression.^5, 6, 7^ Previous reports, combined with our findings, suggest that tumor-associated macrophages (TAMs) mainly exhibit an M2-like phenotype and are associated with tumor angiogenesis, metastasis, and poor prognosis in many human cancers.^8, 9, 10, 11, 12, 13, 14, 15^ Gaining better insight into the origin and regulation of macrophage polarization will provide the means to selectively target or reprogram TAMs so as to impede cancer progression and improve the efficacy of anticancer therapy.^16^

In addition to the high rate of cell proliferation, cell loss in malignant disease is a significant component of tumor dynamics and apoptosis is a common process in high-grade malignancy, with high apoptotic indices generally reflecting poor prognosis.^17^ Indeed, the recognition and removal of apoptotic cells (ACs) by tissue macrophages, a process called efferocytosis, are critical for development and resolution of inflammation.^18, 19, 20^ Recent studies have demonstrated that efferocytosis of ACs induces the transcription of wound-healing cytokines that promote resolution of acute inflammation and tissue repair,^21, 22, 23, 24^ which can elicit M2 macrophage polarization and enhance tumor metastasis.^25, 26^ Despite these links between efferocytosis and macrophage polarization, the specific molecules that mediate these processes remain to be studied.

Intercellular cell adhesion molecule-1 (ICAM-1) is a structurally related transmembrane glycoprotein member of the immunoglobulin supergene family and is the ligand for *β*2 integrin molecules present on leukocytes.^27, 28^ In general, ICAM-1 has a key role in inflammatory conditions, immune responses through antigen recognition, and lymphocyte circulation and activation.^29, 30^ ICAM-1 is involved in signal transduction across cell membranes; leukocyte–leukocyte, leukocyte–endothelial, and leukocyte–epithelial cell interactions; transendothelial migration; and adhesion-dependent respiratory bursts.^31, 32, 33^ In particular, it has been reported that the engagement of ICAM-1 leads to initiation of intracellular signal transduction, designated 'outside-to-inside signaling' from the cell surface.^28, 34^ A recent study suggests that ICAM-1 has a role as a suppressor of tumor progression, which can sensitize metastatic tumor cells to cytotoxic T lymphocyte-mediated killing by interfering with activation of the PTEN/phosphoinositide 3 kinase (PI3K)/AKT pathway.^35^ In a study of colorectal cancer, clinicopathologic analysis suggested an inverse correlation between ICAM-1 expression and the prognostic value, including lymph node and/or liver metastasis and disease-free survival.^36^ However, its roles in regulating the macrophage phenotype and the mechanism for macrophage polarization in tumor microenvironment remain unclear.

The present study examines the molecular basis of efferocytosis and M2 macrophage polarization in the tumor microenvironment. Our results show that deficiency of ICAM-1 expression in macrophages can contribute to efferocytosis of ACs via inhibition of the PI3K/AKT pathway. ICAM-1 deletion-mediated efferocytosis induces macrophage polarization toward the M2 phenotype, leading to promotion of tumor metastasis. These findings highlight the relevance of ICAM-1 in macrophages as a key mediator of immunologic anti-tumor metastasis.

## Results

### Loss of ICAM-1 expression is associated with malignancy and aggressiveness of human colon cancer

To determine whether ICAM-1 expression was associated with colon cancer in humans, we first performed data mining and analyzed ICAM-1 transcript by using the publicly available Oncomine database. Our analysis revealed a significant upregulation in ICAM-1 mRNA expression in colorectal carcinoma tissues as compared with normal specimens. Supplementary Figure 1a depicts the levels of ICAM-1 transcripts across four independent published microarray studies.^37, 38, 39, 40^ Furthermore, in a meta-analysis of recent gene expression profiling, increased ICAM-1 expression was significantly associated with colorectal carcinoma compared with normal (Supplementary Figure 1b). Notably, the box plot data from pathology subtyping within the Kaiser data set showed a lower log2 median expression of ICAM-1 in distant metastasis-positive colorectal carcinoma samples (0.595) compared with non-distant metastasis (0.782; Supplementary Figure 2). These data indicate that decreased ICAM-1 transcript level significantly correlates with distant metastasis of colorectal carcinoma patients.

**Figure 1:**
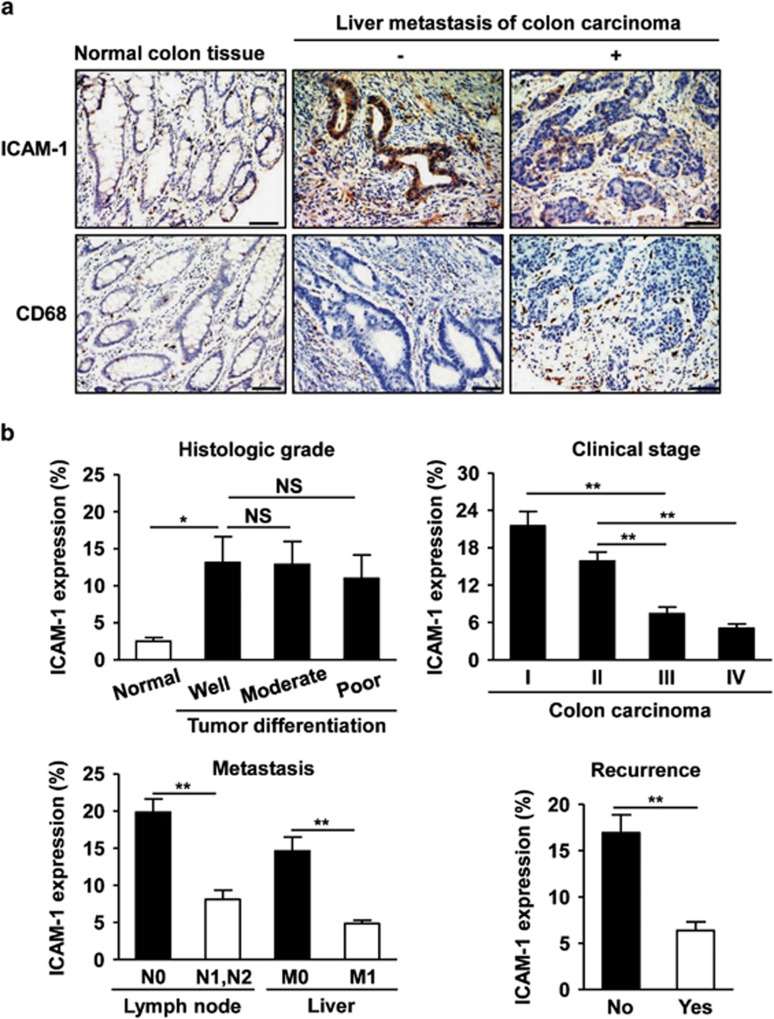
Expression of ICAM-1 in human colon cancer. (**a**) Immunohistochemical staining of ICAM-1 or macrophage marker CD68 in normal colon tissues and human primary colon carcinoma that were classified into liver metastasis-negative group and liver metastasis-positive group ( × 200 magnification and scale bars=100 *μ*m). (**b**) Quantitative analysis of ICAM-1 expression in tumor sections at different histologic grade (well, moderate, and poor), different tumor stages (I–IV), the incidence of lymph node or liver metastasis, and with or without recurrence 3 years after operation (*n*=5–10 samples per group, with 10 fields per samples). N indicates lymph node metastasis. M indicates distant metastasis. *P<0.05, **P<0.01. NS, not significant

To investigate directly whether ICAM-1 expression is associated with the tumor microenvironment and colon carcinoma progression clinically, we used human colon carcinoma and adjacent normal colon tissues to examine ICAM-1 expression concurrently with infiltrating TAMs. Immunohistochemical staining demonstrated that ICAM-1 was weakly detected in normal tissues, and focally in the infiltrating lymphocytes and monocytes of the colon mucosa (Figure 1a). However, a variety of ICAM-1 staining patterns were observed in malignant and metastatic colon cancer. ICAM-1 expression was markedly increased in colon carcinoma, which was mainly distributed not only in the cytoplasm and membrane of tumor cells but also in tumor-infiltrating leukocytes (Figure 1a). Importantly, the expression of ICAM-1 in primary tumors from colon cancer patients with liver metastasis was significantly lower than those of lesions without liver metastasis (Figure 1a). Moreover,the primary tumors with liver metastasis were markedly and extensively infiltrated by CD68-positive macrophages located in the central tumor stroma and at the invasive front of lesions (Figure 1a). A reverse relationship between ICAM-1 expression and macrophage infiltration was observed in comparison to primary colon carcinoma with and without liver metastasis.

The analysis of clinicopathologic parameters showed no differences in ICAM-1 expression with respect to the degree of primary tumor differentiation including well, moderately, or poorly differentiated colon carcinoma (Figure 1b). By contrast, ICAM-1 expression in primary tumors from stage III and IV colon cancer patients was significantly decreased compared with that of primary lesions from stage I and II colon cancer patients (Figure 1b). Moreover, analysis of ICAM-1 expression in primary tumors and metastasis formation revealed that patients who had lymph node or liver metastasis and local recurrence within 3 years showed markedly decreased ICAM-1 expression in colon cancer tissues (Figure 1b). Combined, these data mining and direct expression assessment in human colon cancer suggest that ICAM-1 expression is negatively associated with metastasis and may be a useful indicator of prognosis in patients with colon cancer.

### ICAM-1 deficiency in the host facilitates tumor metastasis

**Figure 2:**
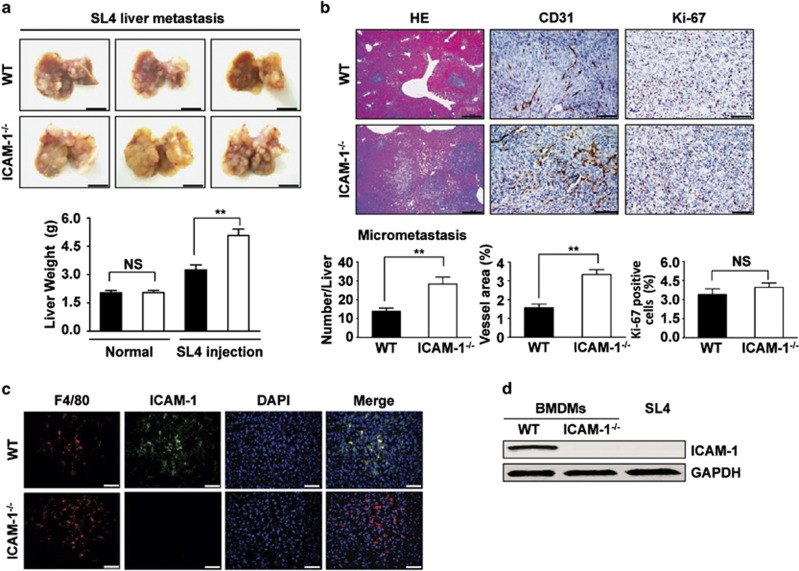
Loss of ICAM-1 in microenvironment aggravates tumor metastasis, promotes tumor angiogenesis, whereas no changes in proliferation of tumor cells. (**a**) Gross examination of development of liver metastasized tumor of colon cancer after intrasplenic injection of SL4 cells in WT and ICAM-1^−/−^ mice. SL4 cells (5 × 10^5^) were injected into the spleen of WT and ICAM-1^−/−^ mice. Mice were killed at 14 days after tumor injection to determine the incidence of liver metastasis and tumor weight. The normal livers from WT or ICAM-1^−/−^ mice as control groups. Data are mean±S.E.M. for *n*=10 mice **P<0.01. NS, not significant. (**b**) Left panel, the analysis of micrometastasis was performed on paraffin-embedded sections with HE staining in metastasized foci after intrasplenic injection of SL4 cells in WT and ICAM-1^−/−^ mice. Number of micrometastatic lesions per one representative cross-section of the livers from each mouse ( × 100 magnification and scale bars=100 *μ*m). Data are mean±S.E.M. for *n*=10 mice with 10 fields per animal. **P<0.01. The area of vessels was evaluated by immunohistochemical analysis with anti-CD31 antibody (middle panel) and the proliferation of tumor cells was examined by immunohistochemical analysis with anti-Ki67 antibody (right panel) in metastasized foci after intrasplenic injection of SL4 cells ( × 200 magnification and scale bars=100 *μ*m). Data are mean±S.E.M. for *n*=8 mice with 10 fields per animal. **P<0.01. NS, not significant. (**c**) Double immunofluorescence analysis of macrophage marker F4/80 and ICAM-1 expression in metastatic foci from WT and ICAM-1^−/−^ mice. The sections were stained with anti-F4/80 (red) or anti-ICAM-1 (green) antibody and DAPI (blue; to stain the nuclei). Scale bars=50 *μ*m. Three independent experiments were performed. (**d**) Western blot analysis of the protein levels of ICAM-1 in BMDMs derived from WT mice or ICAM-1^−/−^ mice, and SL4 cells. GAPDH was used as a loading control. Three independent experiments were performed

To determine whether host-derived ICAM-1 in the tumor microenvironment affects tumor metastasis, we used a colon cancer liver metastasis model, in which SL4 mouse colon cancer cells were injected into the spleens of wild-type (WT) and ICAM-1^−/−^ mice. After 2 weeks, mice were killed; gross inspection showed a marked increase of liver weight in ICAM-1^−/−^ mice because of multiple hepatic tumor nodules and increased tumor-occupied weight compared with that in WT mice, with no difference in normal liver weight between WT and ICAM-1^−/−^ mice (Figure 2a). Hematoxylin and eosin staining showed the number of micrometastatic foci was significantly higher in livers from ICAM-1^−/−^ mice relative to WT mice (Figure 2b). In addition, as shown in Figure 2b, immunostaining for the CD31 antibody (a marker of endothelial cells) demonstrated that the density of CD31-positive microvessels from hepatic metastasized foci was significantly greater in ICAM-1^−/−^ mice than that in WT mice. However, immunohistochemical analysis of the proliferation marker Ki67 showed that ICAM-1^−/−^ mice that developed hepatic metastatic tumors proportionally contained the same levels of Ki67 when compared with metastatic tumors from WT mice (Figure 2b).

To analyze the cellular source of ICAM-1 expression among tumor-infiltrating leukocytes, metastatic foci sections were immunostained with the macrophage marker F4/80 and an anti-ICAM-1 antibody. Immunofluorescence staining showed that ICAM-1 was abundantly expressed in macrophages in hepatic metastatic tumors of WT mice, with no ICAM-1 expression in ICAM-1^−/−^ mice (Figure 2c). Furthermore, western blot was performed in different types of cultured mouse cells. As shown in Figure 2d, ICAM-1 was highly expressed in WT bone marrow-derived macrophages (BMDMs), but scarcely detected in both ICAM-1^−/−^ BMDMs and SL4 cells. These results suggest that ICAM-1 in macrophage host cells plays an important role in tumor metastasis inhibition.

### ICAM-1 deficiency increases M2 macrophage infiltration into the tumor microenvironment

**Figure 3:**
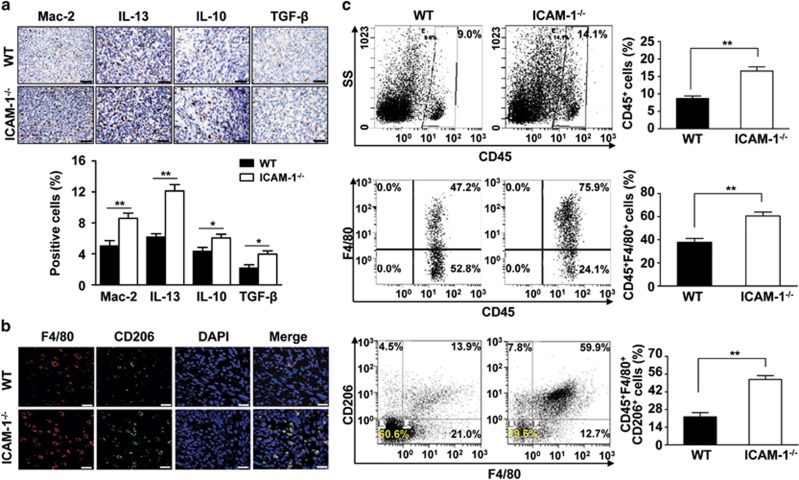
Effects of ICAM-1 on tumor-associated macrophages polarization. (**a**) Immunohistochemical analysis of macrophage infiltration and cytokine expression after intrasplenic injection of SL4 cells in WT and ICAM-1^−/−^ mice. Macrophage infiltration detected by anti-Mac-2 immunostaining ( × 200 magnification and scale bars=100 *μ*m), cytokine expression detected by anti-IL-13, anti-IL-10 and anti-TGF-*β* immunostaining ( × 400 magnification and scale bars=50 *μ*m). Quantitative analysis of macrophage infiltration and cytokine expression in metastasized foci sections. Data are mean±S.E.M. for *n*=8 mice with 10 fields per animal. *P<0.05, **P<0.01 *versus* WT mice. (**b**) Double immunofluorescence analysis of M2 macrophages expression in metastatic foci from WT and ICAM-1^−/−^ mice. The sections were immunostained using the combination of anti-F4/80 (red) or anti-CD206 (green) antibodies and DAPI (blue; to stain the nuclei). Scale bars=50 *μ*m. (**c**) Leukocytes were gated with CD45 fluorescence, macrophages (CD45^+^F4/80^+^cells), macrophage M2 marker (CD45^+^F4/80^+^CD206^+^) were detected by flow from metastatic foci in the liver after intrasplenic injection of SL4 cells in WT and ICAM-1^−/−^ mice. Data are mean±S.E.M. for *n*=8 mice per group. **P<0.01 *versus* WT mice

To determine the contribution of macrophages to tumor development, we characterized cells expressing Mac-2 (the macrophage marker) in both WT and ICAM-1^−/−^ mice. Immunohistochemical staining demonstrated that the expression of Mac-2 in hepatic metastatic tumors was more highly abundant in ICAM-1^−/−^ mice than in WT mice (Figure 3a). Previous studies have shown that tumor-infiltrating macrophages display an M2 phenotype and promote metastasis.^1, 41^ To determine whether ICAM-1 regulates macrophage polarization in the tumor microenvironment, we examined the expression of cytokines in metastatic tumor tissues. We found that the expression of M2-specific cytokines such as interleukin (IL)-13, IL-10, and transforming growth factor-*β* (TGF-*β*) was markedly increased in hepatic metastatic tumors of ICAM-1^−/−^ mice compared with that in WT mice (Figure 3a). Quantitative RT-PCR further confirmed that ICAM-1 deficiency significantly upregulated mRNA levels of IL-10, TGF-*β*, chemokine (C–C motif) ligand 17 (CCL17), CCL22 (M2-specific cytokines or chemokines) in hepatic metastatic tumors (Supplementary Figure 3). Moreover, immunofluorescence analysis showed that macrophages in tumors from ICAM-1^−/−^ mice expressed CD206, an M2 macrophage marker, more strongly than WT mice (Figure 3b).

We further analyzed the macrophage phenotype in metastatic tumor tissues by flow cytometry. Our findings confirmed that CD45^+^F4/80^+^ macrophages were more highly abundant in metastatic tumors in ICAM-1^−/−^ mice than in WT mice (Figure 3c). Importantly, flow cytometry revealed an increased proportion of macrophages that were mainly positive for CD206 expression in ICAM-1^−/−^ mice, and the proportion of CD45^+^F4/80^+^CD206^+^ cells was significantly enhanced in the tumors of ICAM-1^−/−^ mice compared with that of WT mice (Figure 3c). These data suggest that ICAM-1 deficiency may directly activate and modulate macrophage polarization into an M2 type within the tumor microenvironment.

### ICAM-1 deficiency in macrophages leads to promotion of apoptotic tumor cell efferocytosis

To explore the physiologic consequences of loss of ICAM-1 in macrophages, we performed a series of efferocytosis assays to investigate the impact of this protein on the phagocytic capacity of apoptotic tumor cells. SL4 cells were incubated with cisplatin to induce apoptosis, and flow cytometric analysis showed about 60% of cells were apoptotic as verified by annexin V-positive cells (Supplementary Figure 4a). On immunofluorescence staining, ICAM-1 was expressed in WT BMDMs and ICAM-1 expression in WT macrophages was markedly elevated after coculture with apoptotic SL4 cells (Supplementary Figure 4b). Western blotting confirmed that apoptotic SL4 cells significantly increased the level of ICAM-1 protein in WT macrophages (Supplementary Figure 4c).

**Figure 4:**
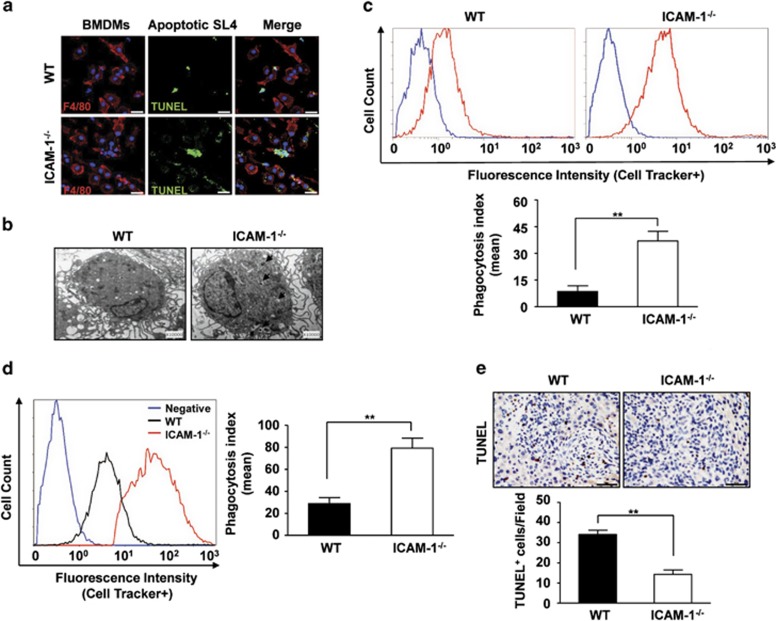
Enhanced efferocytosis of apoptotic tumor cells in ICAM-1^−/−^ macrophages. (**a**) Representative confocal images of stained WT or ICAM-1^−/−^ BMDMs (red) cocultured for 2 h with TUNEL-stained apoptotic SL4 cells (green) at 1 : 5 ratio. Anti-F4/80 staining (red) was used to identify macrophages. TUNEL staining (green) was used to examine apoptosis. DAPI nuclear staining is in blue. Three independent experiments were performed. (**b**) Representative transmission electron micrographs of WT or ICAM-1^−/−^ BMDMs were cocultured with apoptotic SL4 cells for 2 h ( × 10 000 magnification). Three independent experiments were performed. Arrows indicate apoptotic SL4 cells cocultured with macrophages are engulfed and retained in phagsomes. (**c**) Representative flow cytometery data on apoptotic SL4 cells phagocytosis by WT and ICAM-1^−/−^ macrophages *in vitro* after 2 h of incubation with CMFDA-labeled apoptotic SL4 cells at a 1 : 5 (BMDMs/apoptotic SL4 cells) ratio. Background (blue line) represents WT or ICAM-1^−/−^ macrophages culture alone. Data are mean±S.E.M. of three independent experiments. **P<0.01. (**d**) CellTracker Green CMFDA-labeled apoptotic SL4 cells were injected into the peritoneum of WT and ICAM-1^−/−^ mice, and 8 h later, free fluorescent cells in the peritoneal cavity were quantified. Macrophages with engulfed fluorescent apoptotic SL4 cells were quantified by flow cytometery. The phagocytic index was determined as described in Methods. Data are mean±S.E.M. for *n*=8 mice per group. **P<0.01. (**e**) TUNEL analysis in metastatic foci from WT and ICAM-1^−/−^ mice. Average TUNEL^+^ cells per field ( × 400 magnification and scale bars=50 *μ*m). Data are mean±S.E.M. for *n*=8 mice with 10 fields per animal. **P<0.01

To assess the functional phagocytic activities, we cocultured apoptotic tumor cells with WT and ICAM-1^−/−^ BMDMs and engulfment of fluorescent ACs was determined. Fluorescence microscopy demonstrated that TUNEL-positive SL4 cells were present within the cytoplasmic vacuoles of WT macrophages, which was further enhanced in ICAM-1^−/−^ macrophages (Figure 4a). Using transmission electron microscopy (TEM), we confirmed that significantly more efferocytic phagosomes containing AC remnants were identified after coculture of ICAM-1^−/−^ macrophages, whereas there was scarcely any formation of large phagosomes in WT macrophages (Figure 4b). To determine the role of ICAM-1 in phagocytosis by macrophages, WT or ICAM-1^−/−^ BMDMs were cocultured with green CMFDA-FITC-labeled apoptotic SL4 cells, and ingestion of these fluorescent cells was quantified by flow cytometry. As shown in Figure 4c, WT BMDMs engulfed the FITC-labeled apoptotic SL4 cells, whereas the ability of ICAM-1^−/−^ BMDMs to take up apoptotic SL4 cells was significantly enhanced. ICAM-1^−/−^ macrophages demonstrated significantly higher phagocytosis of apoptotic tumor cells than WT macrophages. To further examine whether ICAM-1 deficiency results in a phagocytosis-enhancing effect *in vivo,* CMFDA-FITC-labeled apoptotic SL4 cells were injected into the peritoneum to evaluate the phagocytic index. We found that peritoneal macrophages in ICAM-1^−/−^ mice showed markedly enhanced phagocytosis of fluorescent apoptotic SL4 cells after 8 h of injection relative to WT mice (Figure 4d). Moreover, TUNEL analysis revealed a significant decrease in the presence of apoptotic nuclei in hepatic metastatic tumors of ICAM-1^−/−^ mice compared with their WT counterparts (Figure 4e). These data are consistent with the role of ICAM-1 in macrophage-mediated efferocytosis of apoptotic SL4 cells, and suggest that lack of ICAM-1 caused a reduction of apoptotic tumor cells during tumor metastasis because of stimulation in efferocytosis.

### The PI3K/AKT pathway mediates ICAM-1-associated modulation of apoptotic tumor cell phagocytosis

**Figure 5:**
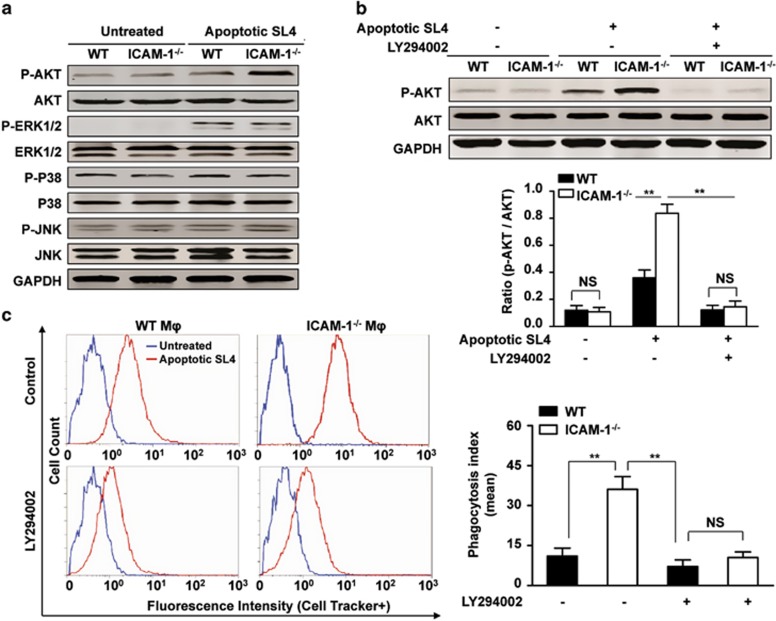
ICAM-1 deficiency contributes to macrophages efferocytosis by activating PI3K/AKT pathway. (**a**) WT or ICAM-1^−/−^ BMDMs were cocultured with apoptotic SL4 cells at a 1: 5 ratio for 30 min. Western blot analysis of the phosphorylation levels of AKT, ERK1/2, P38, JNK in WT or ICAM-1^−/−^ BMDMs by apoptotic SL4 cells coculture. GAPDH was used as a loading control. Three independent experiments were performed. (**b**) WT or ICAM-1^−/−^ BMDMs were pretreated 1 h with the PI3K/AKT inhibitor LY294002 or control DMSO, were then incubated with apoptotic SL4 cells for 30 min. Western blot analysis was performed and phospho-AKT expression levels were normalized to total AKT (P-AKT/AKT). Data are mean±S.E.M. of three independent experiments. **P<0.01, NS, not significant. (**c**) The PI3K/AKT inhibitor LY294002 (10 *μ*mol/l) or DMSO (vehicle) was added to WT or ICAM-1^−/−^ BMDMs for 1 h before coculture with CMFDA-labeled apoptotic SL4 cells and the phagocytic index was measured by flow cytometry. Data are mean±S.E.M. of three independent experiments. M*ϕ* indicates macrophage. **P<0.01. NS, not significant

The mitogen-activated protein kinase (MAPK) signaling pathway has been reported to manipulate phagocytosis.^42, 43^ To elucidate whether the MAPK signaling pathway is involved in the enhanced phagocytic activities of ICAM-1^−/−^ macrophages, we studied the activation of several MAPKs and monitored apoptotic tumor cell phagocytosis. As shown in Figure 5a, no difference in phosphorylation of extracellular signal-regulated kinase 1/2 (ERK1/2), P38, and c-Jun N-terminal kinase (JNK) between ICAM-1^−/−^ and WT macrophages was observed following coculture with apoptotic SL4 cells. By contrast, addition of apoptotic SL4 cells resulted in strong phosphorylation of AKT in ICAM-1^−/−^ macrophages compared with that in WT macrophages (Figure 5a).

To confirm the role of the PI3K/AKT pathway in promoting the phagocytic activities of ICAM-1^−/−^ macrophages, we used a selective pharmacologic inhibitor, the PI3K (the upstream regulator of AKT activation) inhibitor LY294002. Following LY294002 treatment, AKT phosphorylation was significantly inhibited in ICAM-1^−/−^ macrophages primed by feeding with apoptotic SL4 cells (Figure 5b). The phagocytosis index showed that priming of ICAM-1^−/−^ macrophages increased their phagocytic capacity, whereas LY294002 pretreatment of ICAM-1^−/−^ macrophages markedly abrogated the enhanced phagocytosis of apoptotic SL4 cells (Figure 5c). LY294002 treatment of ICAM-1^−/−^ macrophages blocked the effects of the enhanced phagocytic activities similar to WT macrophages treated with LY294002 (Figure 5c). These results demonstrate that ICAM-1 deficiency enhances macrophage phagocytosis via PI3K/AKT pathway activation.

### ICAM-1 deficiency-mediated efferocytosis contributes to M2 macrophage polarization

**Figure 6:**
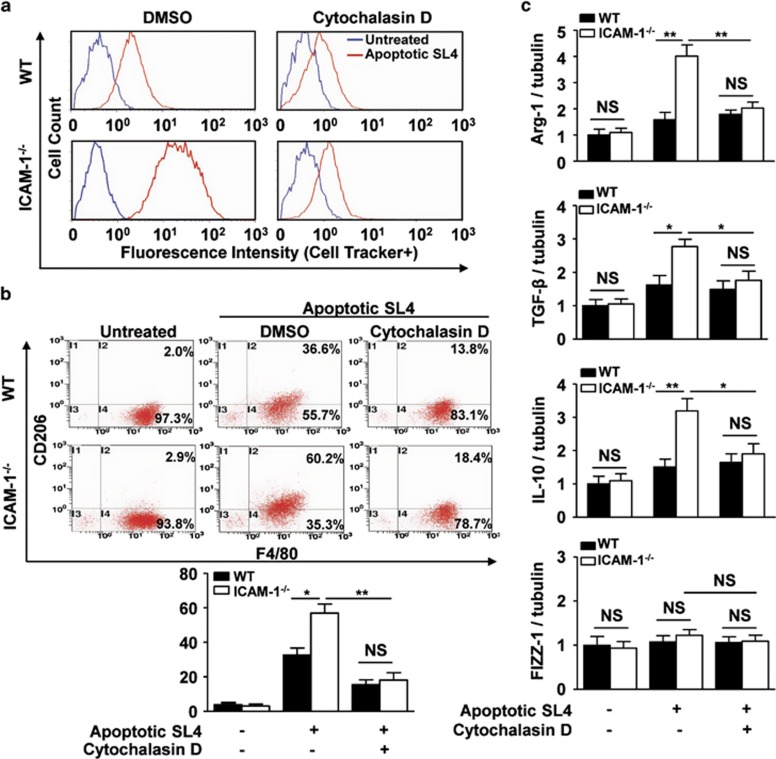
Efferocytosis is required for ICAM-1 deficiency induces M2 macrophages polarization. (**a**) WT or ICAM-1^−/−^ BMDMs were pretreated 1 h with the efferocytosis inhibitor cytochalasin D (10 *μ*mol/l) or control DMSO and then subjected to CMFDA-labeled apoptotic SL4 cells for 2 h, after which the phagocytic index was determined by flow cytometry. Three independent experiments were performed. (**b**) Flow cytometric analysis of M2 macrophages (F4/80^+^CD206^+^) from cocultures treated with or without cytochalasin D (10 *μ*mol/l) for 48 h. Data are mean±S.E.M. of three independent experiments. *P<0.05, **P<0.01. NS, not significant. (**c**) Quantitative real-time PCR analysis of the mRNA expression of M2 marker (Arg-1, TGF-*β*, IL-10, found in inflammatory zone 1 (FIZZ1)) in WT or ICAM-1^−/−^ BMDMs cocultured with apoptotic SL4 cells for 24 h in the presence or absence of cytochalasin D. Tubulin was a normalization control. Data are mean±S.E.M. of three independent experiments. *P<0.05, **P<0.01. NS, not significant

To determine whether ICAM-1 deficiency-mediated efferocytosis is a critical driving force for M2 macrophage polarization, WT or ICAM-1^−/−^ BMDMs were pretreated with cytochalasin D, a potent inhibitor of phagocytosis,^44^ followed by treatment of cocultures with CMFDA-FITC-labeled apoptotic SL4 cells to analyze the macrophage phenotype. As shown in Figure 6a, cocultures pretreated with cytochalasin D displayed impaired uptake of fluorescent apoptotic SL4 cells, confirming the role of cytochalasin D in blocking efferocytosis. Flow cytometric analysis showed that only a small population of M2 macrophages (CD45^+^F4/80^+^CD206^+^ cells) were detected when WT or ICAM-1^−/−^ macrophages were cultured alone (Figure 6b). However, ICAM-1^−/−^ macrophages showed a marked increase in the population of M2 macrophages (CD45^+^F4/80^+^CD206^+^ cells) compared with that in WT macrophages following coculture with apoptotic SL4 cells, which was abolished by cytochalasin D treatment similar to the results observed in WT macrophages (Figure 6b). To further elucidate whether efferocytosis of apoptotic tumor cells has a role in M2 polarization, the expression of M2- and M1-type genes were assayed. ICAM-1^−/−^ macrophages exhibited upregulated mRNA levels of arginase-1 (Arg-1), transforming growth factor-*β* (TGF-*β*) and IL-10 (M2 type genes) as compared with the levels detected in WT macrophages following coculture with apoptotic SL4 cells; pharmacologic inhibition of efferocytosis using cytochalasin D resulted in decreased Arg-1, TGF-*β*, and IL-10 mRNA levels in ICAM-1^−/−^ macrophages, and this profile was similar in WT macrophages following cytochalasin D treatment (Figure 6c). By contrast, M1-type genes inducible nitric oxide synthase (iNOS), tumor necrosis factor-alpha (TNF-*α*), and chemokine (C–X–C motif) ligand 9 (CXCL9) were not significantly changed between WT and ICAM-1^−/−^ macrophages cocultured with apoptotic SL4 cells (Supplementary Figure 5). These data suggest that ICAM-1 deficiency-dependent efferocytosis of apoptotic tumor cells enhances the acquisition of an M2-like phenotype in macrophages.

## Discussion

Effective recognition and clearance of ACs by macrophages are critically important in efferocytosis, and this important endogenous mechanism of controlling the immune response is exploited by pathogens and tumors.^17, 45, 46^ Macrophages have important functions in immune responses and adopt various phenotypes, mainly depending on the environment including the secretion of tumor-derived mediators, hypoxic and necrotic factors, tissue damage, as well as influences from other immune cells and stromal components.^1, 2, 3, 4^ Although there is a growing body of literature on the role of macrophages in tumor progression, little attention has been given to the phagocytic function of macrophages in the tumor microenvironment and the mechanisms that control this process. In this study, the interactions between macrophages and tumor cells were investigated, with focus on efferocytosis and its effects on macrophage polarization via ICAM-1 deficiency.

Recently, variation of expression of several adhesion molecules has been found to influence not only the metastatic cascade, but also escape from immune surveillance.^35^ Particularly, ICAM-1 is a member of the immunoglobulin superfamily of proteins expressed in all leukocytes and on the surface of many cancer cell types, which shows altered expression in malignant diseases and involved in the process of cancer metastasis.^47^ In this study, decreased expression of ICAM-1 in colon cancer could be related to the aggressive nature of the tumor, and has a poor prognostic effect on colon cancer. The same conclusion regarding prognostic was demonstrated previously, as the incidence of lymph node and liver metastasis was significantly lower in colorectal cancer patients with ICAM-1-positive tumors than in those with ICAM-1-negative tumors.^36^ Moreover, clinical research has shown that the loss of ICAM-1 expression is associated with an increased risk of metastasis within the first 5 years after diagnosis of uveal melanoma.^48^ The findings indicate that ICAM-1 may have important clinical significance for assessing condition, curative effect, recurrence, and prognosis in colon cancer patients.

Previous studies underscore the pivotal role of ICAM-1 in the rejection of immunogenic tumors but not for clearance of systemic infections,^49^ suggesting their particular role in anti-tumor immunity. In this study, further promotion of the tumor metastasis in ICAM-1^−/−^ mice compared with WT mice was observed. This suggests a possibly predominant contribution of stroma-derived ICAM-1 as a tumor metastasis suppressor. It may further indicate that loss of ICAM-1 may be required for the pathophysiology of cancer metastasis in the tumor microenvironment. Here, we provide evidence that ICAM-1 deficiency accelerates macrophage infiltration and contributes to macrophage polarization toward a pro-tumor M2 phenotype, implying that endogenous ICAM-1 exerts a metastasis suppressor effect in part through inhibiting macrophage recruitment and M2 polarization in the tumor microenvironment. Thus, it is of great interest to explore the mechanisms by which ICAM-1 modulates macrophage polarization.

Several mechanisms have been reported that may contribute to the development of M2 macrophages, including ingestion of ACs.^17, 45^ The immune system, including integrin-based systems, scavenger receptors, immunoglobulin superfamily molecules, and complement receptors, has been implicated in the clearance of ACs by macrophages.^50^ An increasingly wide variety of intermediate factors are emerging, with the role of creating molecular bridges between components of the AC and phagocyte surfaces. For example, milk fat globule-EGF factor 8 is a protein secreted by numerous cells including macrophages, which functions as a tether between macrophages and ACs via a bi-motif function, binding to both phosphatidylserine externalized on ACs and the *α*v*β*3/*α*v*β*5 integrin expressed on macrophages.^51^ Molecules that bind these integrins, such as extracellular matrix ligands, inhibitory antibodies, and high-mobility group box 1, have also been shown to inhibit efferocytosis.^52, 53^ In the present study, we found that ICAM-1 has a novel role in the efferocytosis of apoptotic tumor cells, acting as a 'do not eat ACs' signal for molecular bridging intermediates of macrophages. Other studies have demonstrated that efferocytosis of dying neutrophils or injured cardiomyocytes, liver cells, or lung epithelial cells induces the transcription of wound-healing cytokines that promote resolution of acute inflammation and tissue repair.^21, 22, 23, 54, 55^ Moreover, efferocytosis is known to produce a shift in macrophage phenotypes toward M2-like characteristics and to induce transcription of Th2-like cytokines, including IL-10 and TGF-*β*.^22, 56^ We show herein that blockade of ICAM-1 deficiency-mediated efferocytosis with a pharmacologic inhibitor can attenuate macrophages toward M2 polarization in the tumor microenvironment. Our findings, together with previous studies, suggest that ICAM-1 is in large part responsible for the suppressed tumor metastasis and decreasing M2-like macrophage presence via abrogation of efferocytosis of apoptotic tumor cells. These findings are consistent with previous reports indicating a critical role for efferocytosis in driving stromal wound-healing and remodeling events that contribute to malignant severity in postpartum breast cancers.^25^

Phagocytosis is controlled by a complex network of signaling pathways. ERK1/2 has been shown to be indispensable for neutrophil phagocytosis, acting downstream of the tyrosine kinase Syk, which is crucial for cytoskeletal rearrangements.^57^ A PI3K-dependent signal transition of Rho-family GTPase activities occurs during FcR-mediated phagocytosis and PI3K-dependent deactivation of Cdc42 at the signal transition is necessary for phagocytosis.^58^ Our findings suggest that ICAM-1 inhibits tumor metastasis through impairing efferocytosis, which is involved in blockade of PI3K/AKT signaling.

In conclusion, we report a novel mechanism by which ICAM-1 in the tumor microenvironment, via restraining efferocytosis of apoptotic tumor cells, can block M2 macrophage polarization through regulation of PI3K/AKT activation, which leads to prevention of tumor metastasis. Our findings on the role of macrophage ICAM-1 in efferocytosis will provide a new therapeutic strategy for cancer metastasis.

## Materials and Methods

### Antibodies and reagents

The antibodies for ICAM-1, CD68, CD206, Mac-2, IL-13, IL-10, TGF-*β*, GAPDH, and IgG were from Santa Cruz Biotechnology (Santa Cruz, CA, USA), the phospho-AKT, AKT, MAPK family antibody sampler kit, phospho-MAPK family antibody sampler kit were from Cell Signaling Technology (Beverly, MA, USA), the antibody for F4/80 was from Abcam (Cambridge, MA, USA), and ChemMate TM EnVision System/DAB Detection Kits were from Dako (Glostrup, Denmark). Antibodies for PerCP/Cy5.5-conjugated CD45.2, phycoerythrin (PE)-conjugated F4/80, fluorescein isothiocyanate (FITC)-conjugated F4/80, and isotype control were from BioLegend (San Diego, CA, USA). Cis-platinum (II) diamine dichloride (cisplatin) was from Sigma-Aldrich (St. Louis, MO, USA). DeadEnd fluorometric TUNEL system was from Promega (Madison, WI, USA). FITC Annexin V apoptosis detection kit was from BD Pharmingen (San Diego, CA, USA). CellTracker Green CMFDA (5-chloromethylfluorescein diacetate) was from Molecular Probes (Eugene, OR, USA). Efferocytosis inhibitor, cytochalasin D, was purchased from Tocris Bioscience (Bristol, UK). PI3K/Akt inhibitor LY294002 was obtained from Calbiochem (San Diego, CA, USA).

### Animals

ICAM-1 knockout (ICAM-1^−/−^) mice and WT littermates were used for all experiments. The ICAM-1^−/−^ mouse strain was obtained from Jackson Laboratory (Bar Harbor, ME, USA), was backcrossed onto the genetic background of C57BL/6 for >10 generations. ICAM-1^−/−^ mice were 10–12 weeks old at the beginning of the experiments, matched for age and sex with WT littermates, and kept under specific pathogen-free conditions at the Beijing Anzhen Hospital, Capital Medical University, China. All animal care and experimental protocols complied with the Animal Management Rule of the Ministry of Health, People's Republic of China (documentation no. 55, 2001) and the Guide for the Care and Use of Laboratory Animals published by the US National Institutes of Health (NIH publication no. 85–23, revised 1996) and were approved by the Institutional Animal Care and Use and Committee of Capital Medical University.

### Tumor model

*In vivo* hepatic metastasis model was performed as previously described in our laboratory.^14, 15, 59^ Murine colon cancer cell line, SL4 cells were maintained in DMEM/F12 culture medium, supplemented with 10% FBS in a humidified 37 °C incubator under 5% CO_2_. Briefly, after anesthetizing mice, a transverse incision in the left flank was made, exposing the spleen, then 5 × 10^5^ SL4 cells in 100 *μ*l DMEM/F12 medium were intrasplenically injected with use of a 26-G needle. Fourteen days after inoculation, mice were killed, and the tissues were processed as described below. The liver weights were measured, and the incidence of liver metastasis was examined.

### Human colon carcinoma specimens

The specimens from 30 cases of human colon carcinoma tissue/adjacent normal colon tissues and the clinicopathologic data were obtained from the Second Affiliated Hospital to Nanchang University gastrointestinal tumor bank. The specimens were isolated at the time of surgery, formalin-fixed and paraffin-embedded, and stained with hematoxylin and eosin, then examined by two experienced pathologists. The clinicopathologic stage was determined according to the TNM classification system of the International Union against Cancer. Human specimens use for research had been approved by the Second Affiliated Hospital to Nanchang University Research Ethics Committee.

### Analysis of oncomine data

ICAM-1 expression from independent published microarray studies was extracted from Oncomine database (http://www.oncomine.org). Briefly, *ICAM-1* gene was queried in the database and the results were filtered by selecting colorectal carcinoma relative to normal colon tissue. Eleven publicly available data sets of *ICAM-1* gene expression were selected for the meta-analysis. Data sets were grouped according to distant metastasis and representative box plots shown to illustrate the difference in ICAM-1 mRNA transcription within cohorts. All data are log transformed and median centered. Standardized normalization techniques and statistical calculations are provided on the Oncomine website and published.

### Immunohistochemistry

Immunohistochemistry of paraffin sections involved the Dako ChemMate TM EnVision System (Dako) with primary antibodies for ICAM-1 (1 : 200), CD68 (1 : 200), CD31 (1 : 200), Mac-2 (1 : 300), IL-13 (1 : 300), IL-10 (1 : 300) or TGF-*β* (1 : 300). Negative controls were omission of the primary antibody, goat nonimmune IgG, rabbit nonimmune IgG, rat nonimmune IgG, or secondary antibody only; in all cases, negative controls showed insignificant staining. To verify the genotype and treatment, quantitative histology involved standard procedures, and results were confirmed by independent pathologists blinded to genotype or treatment group. Images were viewed and captured using a Nikon Labophot 2 microscope (Nikon Corp., Tokyo, Japan) equipped with a Sony CCD-IRIS/RGB color video camera (Sony Corp., Tokyo, Japan) attached to a computerized imaging system and analyzed by Image Pro Plus 3.0. (Media Cybernetics, Bethesda, MD, USA). The expression of ICAM-1, macrophage marker, or cytokines was calculated as proportion of positive area to total tissue area for all measurements of the sections.

Frozen tumor sections (7 *μ*m) and cell slides were incubated with the primary antibodies for F4/80 (1 : 100), ICAM-1 (1 : 200), CD206 (1 : 200) at 4 °C overnight and then with FITC- or tetramethylrhodamine isothiocyanate-conjugated secondary antibodies (Jackson ImmunoResearch Laboratories Inc., West Grove, PA, USA) at room temperature for 1 h. Sections were viewed with a confocal laser scanning microscope (TCS 4D; Leica, Heidelberg, Germany) and a Nikon Labophot 2 microscope equipped with a Sony CCD-IRIS/RGB color video camera.

### Flow cytometry

The content of inflammatory cells was quantified by flow cytometry as previously described by us.^15^ Briefly, tumor tissues were cut into multiple small cubes and digested in an enzyme mixture for 45 min at 37 °C. The cell suspension was centrifuged and pre-incubated with Fc-*γ* block antibody (anti-mouse CD16/32; Pharmingen, San Diego, CA, USA) to prevent nonspecific binding. Cell staining involved different combinations of fluorochrome-coupled antibodies to CD45.2, F4/80, CD206 for 30 min at 4 °C in the dark. Fluorescence data were collected by use of an EPICS XL flow cytometer (Beckman Coulter, San Diego, CA, USA) and analyzed using CellQuest (BD Biosciences, San Jose, CA, USA). Fluorescence minus one controls were included to determine the level of nonspecific staining and auto-fluorescence associated with subsets of cells in each fluorescence channel.

### Cell culture

BMDMs were isolated from tibias and femurs of 12-week-old WT and ICAM-1^−/−^ mice as previously described.^60^ To generate ACs, SL4 cells were induced by overnight serum starvation followed by 24 h treatment with Cisplatin (20 *μ*g/ml). Cells were washed at least three times with PBS after apoptosis induction and enumerated, a 60–70% range of annexin V-positive cells were designated ACs.

WT or ICAM-1^−/−^ BMDMs were plated at 50 000 cells/cm^2^, pretreated with the efferocytosis inhibitor cytochalasin D (10 *μ*mol/l) or PI3K inhibitor LY294002 (10 *μ*mol/l) for 1 h, and controls received equivalent dilution with DMSO vehicle alone. Cells were then incubated at different time points with apoptotic SL4 cells at a 1 : 5 ratio of BMDMs to apoptotic SL4 cells. Cells were washed with PBS and attached cells were collected for further analyses.

### TUNEL staining

Identification of apoptosis on SL4 cells grown on coverglass was performed using fluorescent *In situ* Cell Death Detection Kit (Promega). Briefly, SL4 cells plated onto coverglass in the presence of 20 *μ*g/ml Cisplatin for 24 h and fixed with 4% paraformaldehyde. Cells were permeabilized using 0.1% solution of Triton X in 0.1% sodium citrate. Consequently, cells were incubated with TUNEL reaction mixture for 1 h at 37 °C according to the manufacturer's instructions. Nuclei were stained with hematoxylin or DAPI (Invitrogen, Carlsbad, CA, USA).

### Annexin V assay

For confirmation of apoptosis, cells were double-stained with Annexin V FITC and propidium iodide (PI) by using the Annexin V Apoptosis Detection kit (BD Pharmingen), according to the manufacturer's recommendations. For analysis, the cells were divided into four distinct populations using the control cells as a reference: costaining with Annexin V and PI allows differentiation of viable cells (Annexin V^-^, PI^-^) from early ACs (Annexin V^+^, PI^-^) and late ACs (Annexin V^+^, PI^+^).

### Transmission electron microscopy

WT or ICAM-1^−/−^ BMDMs were cultured in six-well dishes and incubated with apoptotic SL4 cells for 2 h, after which the cells were harvested by trypsinization and centrifuged at low speed for 5 min. The resulting cell pellets were fixed with cold 1% glutaraldehyde for TEM assay by routine procedures as previously described.^14^ Samples were embedded, cut on a Reichert Ultracut-S microtome, picked up onto copper grids, and stained with lead citrate and examined using the TEM (Philips EM410) and recorded with an AMT 2k CCD camera.

### Efferocytosis assay

*In vivo* efferocytosis was determined as previously described.^24^ In brief, apoptosis was induced in SL4 cells that were labeled with 10 *μ*mol/l of the green fluorescent marker 5-chloromethylfluorescein diacetate (CellTracker Green CMFDA, Molecular Probes) according to the manufacturer's instructions. CellTracker Green CMFDA-labeled apoptotic SL4 cells (1 × 10^7^ cells) were injected intraperitoneally into WT and ICAM-1^−/−^ mice. After 8 h, mice were killed and peritoneal lavage performed using sterile PBS containing EDTA (5 *μ*mol/l). Isolated cells were washed with culture media and then incubated in PBS containing 1% albumin, stained with PE-conjugated anti-F4/80 (macrophage marker) antibody followed by flow cytometry analysis.

Assays for *in vitro* efferocytosis of ACs were performed as previously described.^61^ WT or ICAM-1^−/−^ BMDMs were pretreated with cytochalasin D (10 *μ*mol/l) or LY294002 (10 *μ*mol/l) for 1 h, followed by CellTracker Green CMFDA-labeled apoptotic SL4 cells coculture at a ratio of 1 : 5. After 2 h of coculture, the cells were harvested, washed, incubated with PE-conjugated anti-F4/80 antibody, and analyzed by flow cytometry. The percentage of F4/80^+^ macrophage that have internalized green apoptotic material detected as double-positive cells was obtained. Results for phagocytosis index are expressed as the percentage of macrophages that are positive for CellTracker Green CMFDA staining.

In selected experiments, 5 × 10^6^ apoptotic SL4 cells were cocultured with 1 × 10^6^ macrophages on glass coverslips for 2 h. Next, coverslips were washed three times with ice-cold PBS and identification of ACs was performed using TUNEL staining. After blocking with 10% normal goat serum, coverslips were incubated with 1 : 100 rat anti-mouse ICAM-1 mAbs for 1 h at room temperature. After washing, visualization was achieved by incubating the coverslips with goat anti-rat Alexa Fluor 488 (Molecular Probes) for 30 min at room temperature. Coverslips were washed, mounted, and examined with a LeicaDM6000 fluorescence microscope (Leica Microsystems). Phagocytosis was evaluated by a blinded observer by counting for five to six randomly selected fields per slide, and calculated as the percentage of macrophages containing at least one engulfed ACs.^62^

### Real-time RT-PCR

Total RNA was prepared using TRIZOL (Invitrogen), and first-strand cDNA was synthesized with use of Superscript II (Invitrogen). Real-time PCR was performed using the SYBR Green Mix (Bio-Rad, Hercules, CA, USA) on the CFX96 Real-time System (Bio-Rad) in duplicate. The primer sequences were as follows:


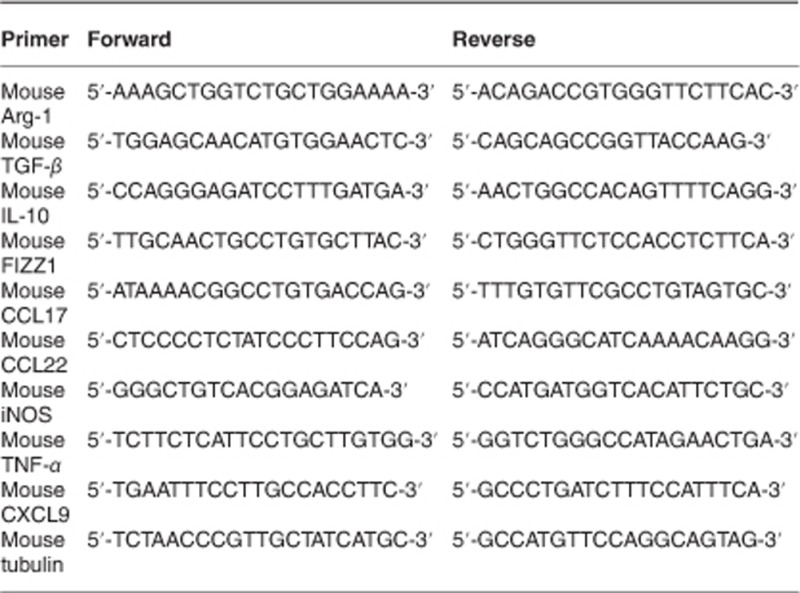


### Western blotting

Protein extracts were diluted with loading buffer and separated by electrophoresis on 8–10% SDS-polyacrylamide gels before transfer to nitrocellulose membranes (Bio-Rad). The membranes were blocked in Odyssey blocking buffer (LI-COR Bioscience, Lincoln, NE, USA) at room temperature for 1 h, then incubated at 4 °C overnight with primary antibodies: ICAM-1 (1 : 800), phospho-AKT (1 : 1000), AKT (1 : 1000), phospho-ERK1/2 (1 : 1000), ERK1/2 (1 : 1000), phospho-P38 (1 : 1000), P38 (1 : 1000), phospho-JNK (1 : 1000), JNK (1 : 1000), GAPDH (1 : 3000). The membranes were washed three times in TBST and incubated with fluorescent secondary antibodies (Alexa Fluor 680 or IRDye 800, Rockland Immunochemicals, Gilbertsville, PA, USA) for 1 h at room temperature at 1 : 5000, blots were analyzed with the Odyssey infrared imaging system and Odyssey software.

### Statistical analysis

Data analysis involved use of GraphPad software (GraphPad Prism version 5.00 for Windows, GraphPad Software, San Diego, CA, USA). Results are expressed as mean±S.E.M. Differences were analyzed by *t*-test or ANOVA, and results were considered significant at *P*<0.05.

## Abbreviations

TAMs: tumor-associated macrophages
ICAM-1: intercellular cell adhesion molecule-1
PI3K/AKT: phosphatidylinositol 3 kinase/Akt
BMDMs: bone marrow-derived macrophages
TEM: transmission electron microscopy
IL: Interleukin
MAPK: mitogen-activated protein kinase
ERK1/2: extracellular signal-regulated kinase 1/2
JNK: c-Jun N-terminal kinase
Arg-1: arginase-1
TGF-*β*: transforming growth factor-*β*
FIZZ1: found in inflammatory zone 1
CCL: chemokine (C–C motif) ligand
iNOS: inducible nitric oxide synthase
TNF-*α*: tumor necrosis factor-alpha
CXCL9: chemokine (C–X–C motif) ligand 9
