# Supplementary material for: ICAM-1 suppresses tumor metastasis by inhibiting macrophage M2 polarization through blockade of efferocytosis
Source: Cell Death Dis. 2015 Jun 11;6(6):e1780–. doi: 10.1038/cddis.2015.144 (PMC4669827; doi:10.1038/cddis.2015.144)
Supplement: Supplementary Information [file cddis2015144x1.pdf]

Supplementary Figure 1

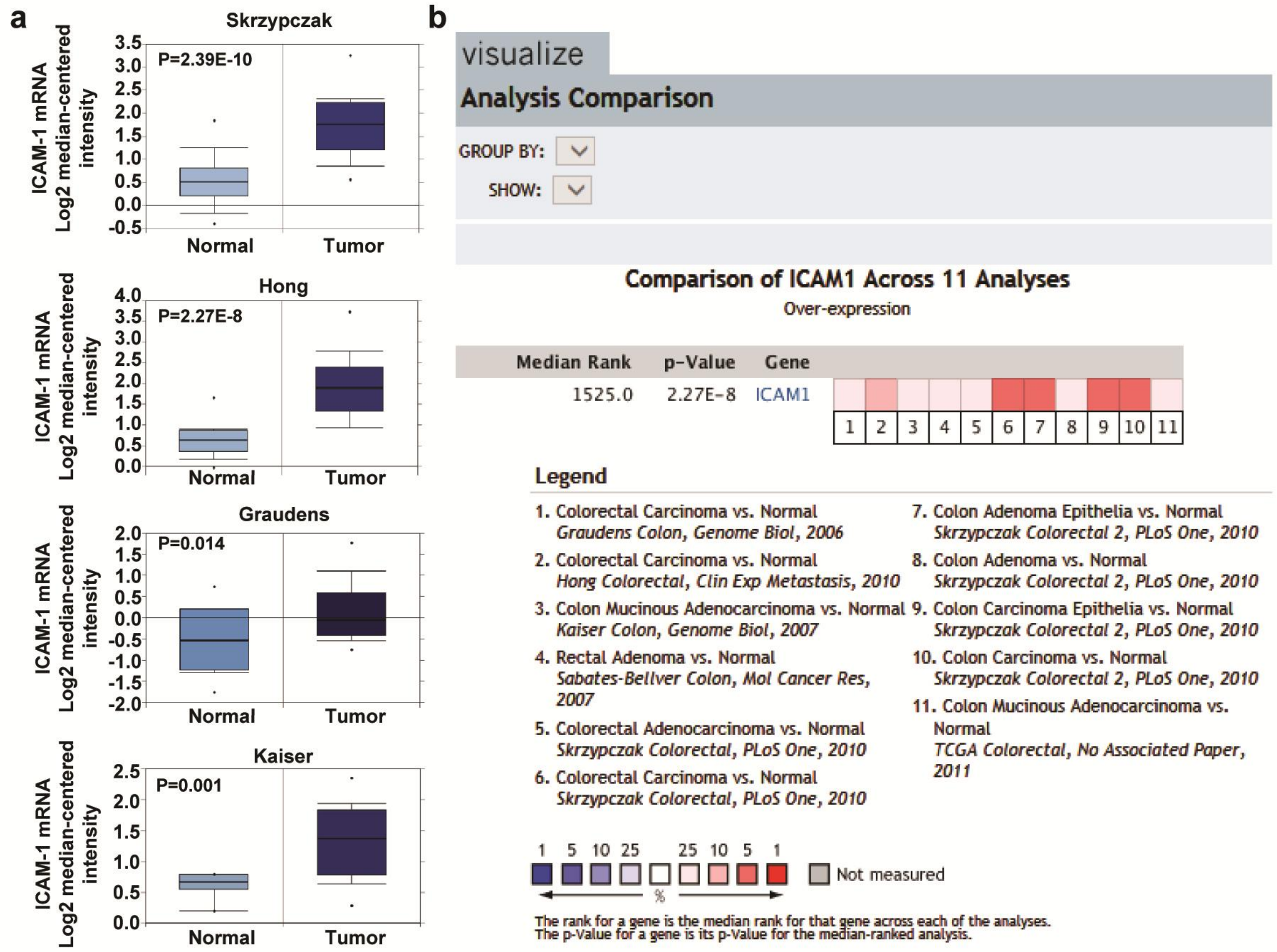

Supplementary Figure 1. Analysis of ICAM-1 expression in human colorectal carcinoma compared with normal tissues by using Oncomine database. (a) Boxplots representation of ICAM-1 mRNA expression in four independent colorectal carcinoma microarray datasets that were identified using filters (Analysis type: Cancer vs. Normal and Cancer type: colorectal carcinoma) and thresholds (P-value=0.05; fold change=2; gene rank=top 10%). All data are log transformed and median centered. First author and statistical significance are indicated. **(b)** Meta-analysis of recent gene expression profiling for ICAM-1 where the colored squares indicate the median rank for ICAM-1 across each analysis.

Supplementary Figure 2

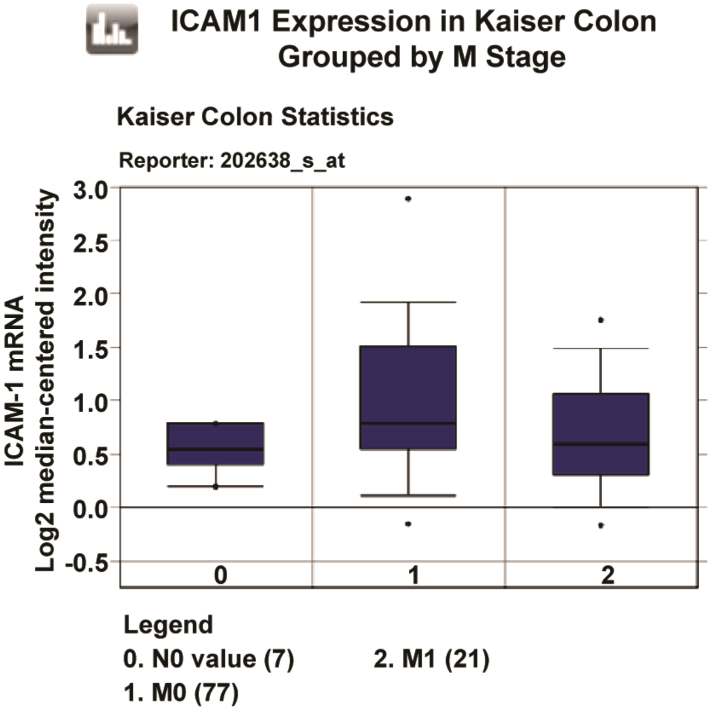

Supplementary Figure 2. Downregulation of ICAM-1 in colorectal carcinoma correlates with patients' distant metastasis. Oncomine database was used for analysis and visualization. M indicates distant metastasis. All data are log transformed and median centered. The 25th–75th percentiles are indicated within the closed blue box; the median is indicated by the solid line; the 10th and 90th percentiles are indicated by the bars. Closed circles above and below the plots show sample maximum and minimum values. The number of colorectal carcinoma samples present in each group is shown within parentheses. Analysis using available clinicopathologic data from the Kaiser colon dataset revealed significant correlation between decreased ICAM-1 level and the distant metastatic status of patients with colorectal carcinoma.

Supplementary Figure 3

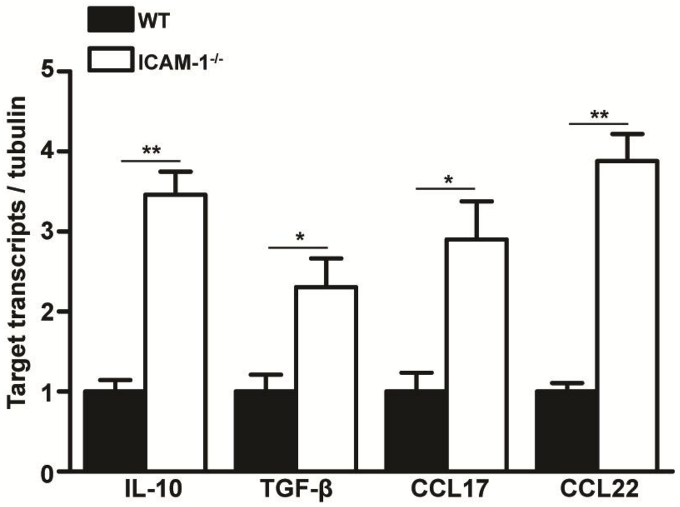

Supplementary Figure 3. ICAM-1 deficiency increases the M2-specific cytokines or chemokines in hepatic metastatic tumors. Quantitative real-time polymerase chain reaction (PCR) analysis of the mRNA level of IL-10, TGF-β, CCL17 and CCL22 in metastatic foci from WT and ICAM-1$^{-/-}$ mice. Tubulin was a normalization control. Data are mean±S.E.M. for n=8 per group. *, *P*＜0.05, **, *P*＜0.01 vs WT mice.

Supplementary Figure 4

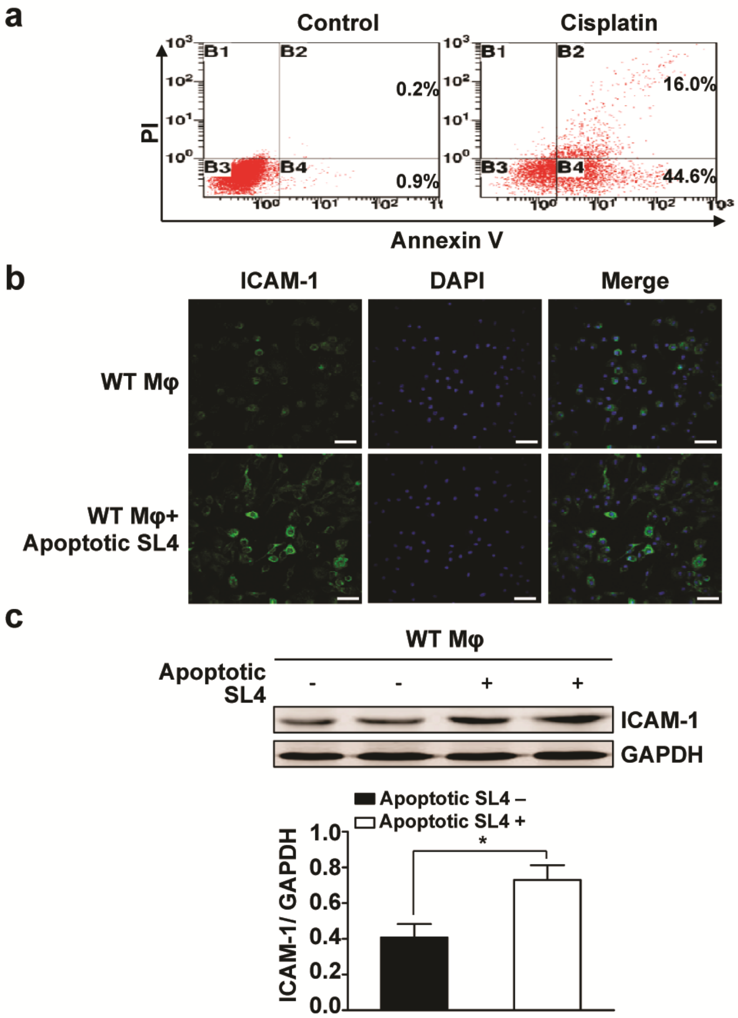

Supplementary Figure 4. Apoptotic SL4 cells induce ICAM-1 expression in macrophages. (a) SL4 cells were treated with cisplatin (20 μg/ml) for 24 h in a serum-free medium after which, apoptosis was determined by flow cytometry using annexin V/propidium iodide staining. Three independent experiments were performed. **(b)** WT BMDMs were cocultured with apoptotic SL4 cells for 24 h, double immunofluorescence analyses of ICAM-1 expression in WT BMDMs with or without apoptotic SL4 cells treatment. BMDMs were stained with anti-ICAM-1 (green) antibody and DAPI (blue; to stain the nuclei). (Scale bars = 25 μm). Three independent experiments were performed. **(c)** Western blot analysis of the protein levels of ICAM-1 in WT BMDMs with or without apoptotic SL4 cells treatment. GAPDH was used as a loading control. Data are mean±SEM of 3 independent experiments. Mφ indicates macrophage. *, $P < 0.05$.

Supplementary Figure 5

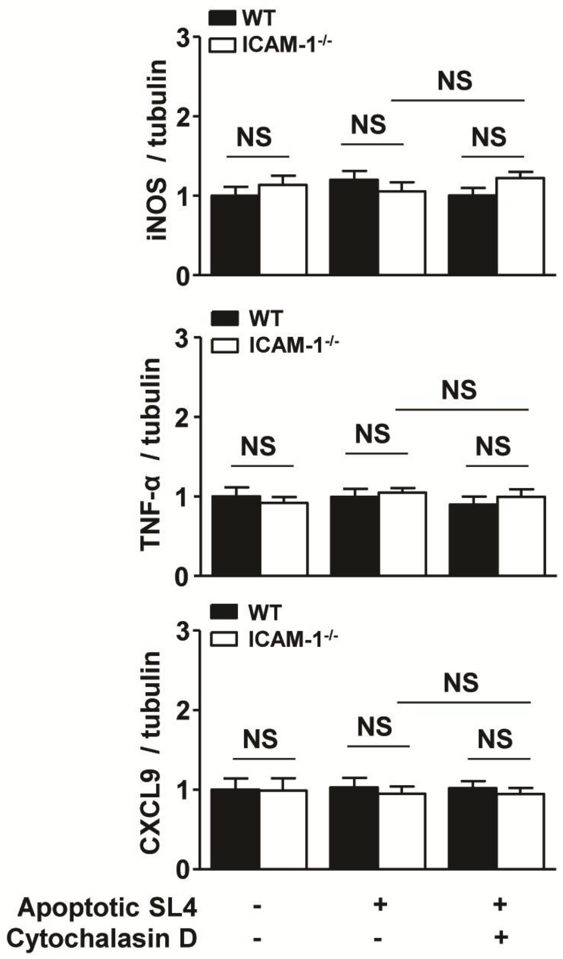

Supplementary Figure 5. Effects of ICAM-1 on the M1 gene profile. WT or ICAM-1$^{-/-}$ macrophages were pretreated with 10 μmol/l efferocytosis inhibitor (cytochalasin D) or DMSO (vehicle) for 1 h and then subjected to apoptotic SL4 cells coculture for 24 h. Quantitative real-time PCR analysis of the mRNA expression of M1 marker (iNOS, TNF-α, CXCL9). Tubulin was a normalization control. Data are mean ± SEM of 3 independent experiments. NS indicates not significant.
